# Shenqi compound enhances pancreatic β-cell secretion by promoting the maturation and transport of insulin secretory vesicles through the NOD1/RIP2 signaling pathway

**DOI:** 10.3389/fnut.2025.1690849

**Published:** 2026-01-26

**Authors:** Nairong Yao, Yiqian Xing, Ruobing Tang, Chunguang Xie, Qiyue Yang, Ya Liu, Xiyu Zhang

**Affiliations:** 1 Department of Endocrinology, Hospital of Chengdu University of Traditional Chinese Medicine, Chengdu, Sichuan, China; 2 Department of Traditional Chinese Medicine, Qingdao Central Hospital, University of Health and Rehabilitation Sciences, Qingdao, Shandong, China; 3 TCM Regulating Metabolic Diseases Key Laboratory of Sichuan Province, Hospital of Chengdu University of Traditional Chinese Medicine, Chengdu, Sichuan, China

**Keywords:** Shenqi compound, pancreatic β-cells, insulin secretory vesicles, insulin secretion, NOD1/RIP2 signaling pathway

## Abstract

**Background:**

Shenqi compound (SQC) is an effective prescription in Chinese medicine to enhance glucose homeostasis and protect pancreatic cells from high glucose-induced damage. However, the protection mechanism remains unclear.

**Objective:**

To investigate the effect of SQC on INS-1 cell secretion and evaluate the associated mechanisms.

**Methods:**

INS-1 cells were cultured in serum augmented with or without NOD1 inhibitor ML130 (2μM) for 1 h, then exposed into a high glucose (50 mM) condition to simulate type 2 diabetes mellitus (T2DM) for 24 h and treated with different concentrations (0, 5, 10, 15, 20%) of SQC in serum for another 24 h. Then, the cell counting kit-8 (CCK-8) method, glucose-stimulated insulin secretion (GSIS) assay, transmission electron microscopy (TEM), real-time fluorescence quantitative reverse transcription polymerase chain reaction (RT-qPCR), and Western blot were performed for further investigation.

**Results:**

Under high glucose conditions, 15% SQC was the optimal therapeutic concentration, significantly improved INS-1 cell viability (*p* = 0.032) and enhanced insulin secretion (*p* < 0.0001). Ultrastructural analysis showed that after high glucose stimulation in GSIS, especially at 20 min, 15% SQC significantly increased both the total density (*p* = 0.143) and the mature ratio (*p* = 0.003) of insulin secretory vesicles. Furthermore, 15% SQC facilitated the dynamic transport of vesicles toward the cell membrane, evidenced by an increased vesicle density within 300 nm of the membrane at 10 min, followed by a subsequent decrease at 20 min—a trend consistent with that observed in the control group. Moreover, at the molecular level, 15% SQC intervention markedly up-regulated NOD1 and RIP2 protein expression (*p* = 0.029 and *p* < 0.0001) and transcription (*p* = 0.886 and *p* = 0.393) levels, while ML130 reversed the activation of the NOD1/RIP2 pathway.

**Conclusions:**

SQC promotes the maturation and transport of insulin secretory vesicles, thereby enhancing the secretory function of INS-1 cells in response to high glucose-induced damage. This protective effect may be associated with the activation of the NOD1/RIP2 signaling pathway.

## 1 Introduction

Type 2 diabetes mellitus (T2DM) is a complex metabolic disorder characterized by chronic hyperglycemia resulting from pancreatic β-cell dysfunction and insulin resistance, leading to multisystem complications and long-term organ damage (1–3). It has emerged as a significant global public health challenge. According to data from the 11th edition of the Global Diabetes Map, approximately 589 million adults aged 20–79 years were living with DM in 2024, a number projected to rise to 853 million by 2050, with over 90% of cases attributed to T2DM (4). Insulin, synthesized and secreted by pancreatic β-cells, is the only hormone in the body that exerts hypoglycemic effects. Consequently, the loss of β-cell mass and function is strongly associated with the development of T2DM (5). Although various oral and injectable glucose-management medicines exist, their efficacy in improving β-cell dysfunction remains limited. Therefore, investigating potential and efficient strategies to protect β-cells is a promising therapeutic avenue for T2DM.

Shenqi compound (SQC), a traditional Chinese medicine formulation effective in the management of T2DM, has been clinically prescribed for over two decades (6). Previous clinical trials revealed that SQC had positive curative effect outcomes. It substantially decreased fasting and postprandial blood glucose levels in T2DM patients, as well as improved pancreatic β-cell function and insulin resistance (7). Furthermore, animal studies have revealed that SQC protects pancreatic β-cell secretory function through multiple pathways, including attenuation of oxidative stress and inflammatory responses, inhibition of pancreatic β-cell hypertrophy, apoptosis, and senescence, and enhancement of glucagon-like peptide-1 (GLP-1) production (8–10). As mentioned above, both clinical evidence and research findings indicate that SQC is a promising hypoglycemic agent with the capacity to preserve pancreatic β-cell function. However, the precise mechanism underlying SQC's protective effects on pancreatic β-cells remains unclear, which limits its broader clinical application. Therefore, it is imperative to elucidate the specific mechanisms by which SQC maintains β-cell secretory function.

Nucleotide-binding oligomerization domain receptor 1 (NOD1) is an essential member of the intracellular pattern recognition receptors (PRRs) family and plays a key role in mediating the innate immune response. According to research, NOD1 activation exerts a bidirectional regulatory effect on systemic glucose homeostasis (11). On the one hand, NOD1 can recognize a variety of ligands and signals derived from exogenous microorganisms, endogenous by-products, and metabolic cues, such as glucose and fatty acids (12, 13). It subsequently recruits receptor-interacting protein 2 (RIP2), activates the nuclear factor κB (NF-κB) and mitogen-activated protein kinase (MAPK) signaling pathways, and then induces inflammatory responses and insulin resistance in key insulin-sensitive metabolic tissues (e.g., liver, muscle, adipose tissue, heart, and kidneys) as well as in circulating immune cells (e.g., monocytes and macrophages), thereby promoting the development of T2DM (14–18). On the other hand, under normal physiological conditions, NOD1/RIP2 signaling is essential for insulin transformation and intracellular distribution (19). Ligands derived from gut microbiota activate NOD1/RIP2 in pancreatic β-cells, leading to the recruitment of Ras-related protein Rab-1A (Rab1a), which mediates the maturation and intracellular transport of secretory vesicles and facilitates their translocation from the perinuclear region to the cell membrane to enable insulin secretion. Moreover, it has been demonstrated that both the deficiency of NOD1 ligands such as γ-D-Glu-meso-diaminopimelic acid (iE-DAP) and the absence of NOD1 in β-cells can impair insulin maturation and transport. Since NOD1/RIP2 signaling is associated with insulin sensitivity and secretion, this complex interaction is critical for protecting pancreatic β-cell function during chronic hyperglycemia.

Based on these findings, this study aims to investigate the bidirectional regulatory effect of SQC on the NOD1/RIP2 signaling pathway in protecting pancreatic β-cell function. Preliminary experiment has shown that in diabetic mice, SQC modulates gut microbiota, repairs the intestinal mucosal barrier, reduces circulating iE-DAP levels, and inhibits the activation of the NOD1/RIP2 and downstream MAPK signaling in pancreatic tissue, thereby preserving β-cell function (20). This study will further examine whether SQC can activate the NOD1/RIP2 pathway in pancreatic β-cells within a diabetic environment under conditions of NOD1 ligand deficiency, and promote insulin vesicle maturation and trafficking, thereby enhancing secretory function.

## 2 Materials and methods

### 2.1 Preparation of SQC

SQC consists of *Panax ginseng* C.A.Mey. (Araliaceae), *Astragalus mongholicus* Bunge (Fabaceae), *Trichosanthes kirilowii* Maxim. (Cucurbitaceae), *Rehmannia glutinosa* (Gaertn.) DC. (Orobanchaceae), *Salvia miltiorrhiza* Bunge (Lamiaceae), *Cornus officinalis* Siebold & Zucc. (Cornaceae), *Dioscorea oppositifolia* L. (Dioscoreaceae) and *Rheum officinale* Baill (Polygonaceae). The SQC granules were produced by the Hospital of Chengdu University of Traditional Chinese Medicine (Chengdu, China). All crude herbs were decocted with water, filtered, concentrated, added with the appropriate amount of excipients, dried, mixed, and then granulated, which were prepared into the dispensing granules at a quality ratio of 8.13:1. Table 1 presents detailed information about SQC granules. Pharmacopeia of the People's Republic of China 2020 and https://mpns.science.kew.org/mpns-portal/ were screened to acquire all herbal data.

**Table 1:** Composition of SQC.

**Latin name**	**Herbal scientific name**	**Dose proportion (g)**	**Batch number**
Ginseng Radix et Rhizoma	*Panax ginseng* C.A.Mey.	15	21,050,001
Astragali Radix	*Astragalus mongholicus* Bunge	15	21,100,223
Trichosanthis Radix	*Trichosanthes kirilowii* Maxim.	10	21,070,136
Rehmanniae Radix	*Rehmannia glutinosa* (Gaertn.) DC.	10	21,070,065
Salviae Miltiorrhizae Radix et Rhizoma	*Salvia miltiorrhiza* Bunge	10	21,070,163
Corni Fructus	*Cornus officinalis* Siebold & Zucc.	10	21,050,004
Dioscoreae Rhizoma	*Dioscorea oppositifolia* L.	10	21,050,094
Rhei Radix et Rhizoma	*Rheum officinale* Baill.	6	21,090,100

### 2.2 Preparation of SQC-containing serum

Sprague-Dawley (SD) rats (8-week-old, male, 240 ± 10 g, *n* = 25) were purchased from Chengdu Dossy Experimental Animal Co., Ltd. [SCXK(chuan)2020-0030]. The animal experiment was authorized by the Laboratory Animal Ethics Committee of Chengdu University of Traditional Chinese Medicine (NO.2024159). All animals were housed in the Experimental Animal Research Centre of Chengdu University of Traditional Chinese Medicine [SYXK(chuan)2019-0049] at 22–25 °C, 50-60% humidity, and a 12 h light-dark cycle.

The SD rats were randomly categorized into the SQC (*n* = 10) and negative control (*n* = 15) groups according to weight stratification. The SQC group rats received SQC solution [SQC decoction-free granules (0.95 g/kg/d) mixed with sterile water], whereas the negative control group received an equal amount of sterile water once daily for 7 consecutive days. Detailed information regarding the animal experiments is provided in Table 2. On the last day of the experiment, 2 h after the last gavage, all rats were anesthetized by urethane (1 g/kg; Macklin, China, U820333) (21). The blood of all the rats was collected from the abdominal aorta and centrifuged (4 °C, 3,000 rpm, 10 min) to collect the supernatant, which was then inactivated, sterilized (56 °C, 30 min), filtered (0.22 μm sterile filter membrane), and then stored at −80 °C until use. 100 μL, 200 μL, 300 μL, and 400 μL of SQC-serum were diluted to a final volume of 2 mL with normal rat serum and medium to prepare 5%, 10%, 15%, and 20% drug-containing serum (22, 23). The main components of SQC-serum and blank-serum analyzed by ultra-performance liquid chromatography-tandem mass spectrometry (UPLC-MS) (Thermo, America, Orbitrap Exploris 120) were shown in Supplementary material (24).

**Table 2:** Animal rearing for the preparation of SQC-containing serum.

**Group**	**Animal**	**Sample size**	**Method of administration**	**Drug dose**	**Intervention period**
SQC	SD rats	10	Gavage	SQC decoction-free granules, 0.95 g/kg/d	Once daily, for 7 consecutive days
Control	SD rats	15	Gavage	Sterile water, an equal amount	Once daily, for 7 consecutive days

### 2.3 Cell culture

The rat insulinoma cell line, INS-1, was purchased from the National Resource Bank for Biomedical Experimental Cells, Institute of Basic Medical Sciences, Chinese Academy of Medical Sciences, China (Item No. 2021022517244). The cells were cultured in RPMI-1640 medium (Servicebio, China, G4538) augmented with 1% penicillin-streptomycin solution (Biosharp, China, BL505A), 10% fetal bovine serum (FBS) (Procell, China, 164210), 50 μmol/L 2-mercaptoethanol (Aladdin, China, M657455), and 5.5 mmol/L glucose (BioFroxx, China, 1179GR500) at 37 °C in a 5% CO_2_ incubator (PHC corporation, Japan, MCO-18AC).

### 2.4 Cell viability assay

Cell viability was determined by the cell counting kit-8 (CCK-8) (Beyotime, China, C0037). INS-1 cells (1 × 10^3^/well) were cultured in 96-well plates and then treated with different high glucose or SQC concentrations for the optimal time. Then, 10 μL of CCK-8 solution was added to each well and incubated for 4 h at 37 °C and 5% CO2. The optical density (OD) at 450 nm was measured using a multifunctional enzyme labeling instrument (BIO-TEK, USA, MQX200). Based on the mean OD value of each group, cell survival rate was calculated as follows:


$$\begin{array}{l} \text{ Cell survival rate }\left(\text{\%}\right)=\\ \frac{\text{OD of experimental group}-\text{ OD of blank group}}{\text{OD of normal group }-\text{ OD of blank group}}\times \;100\text{\%} \end{array}$$

### 2.5 Glucose-stimulated insulin secretion (GSIS)

INS-1 cells (5 × 10^3^/well) were cultured in 96-well plates in media augmented with 5.5 or 50 mM glucose for 24 h and then treated with 0, 10, 15, and 20% SQC for another 24 h. The cells were then washed and incubated for 1 h in Krebs-Ringer bicarbonate HEPES buffer [KRBB: 4.8 mM KCl, 129 mM NaCl, 2.5 mM CaCl_2_, 1.2 mM KH_2_PO_4_, 1.2 mM MgSO_4_, 5 mM NaHCO_3_, 0.5% bovine serum albumin (BSA), 20 mM HEPES, pH 7.4]. The buffer was then replaced with a KRBB buffer containing 5.5 or 50 mM glucose for 1 h at 37 °C. The supernatant was collected to measure insulin secretion by enzyme-linked immunosorbent assay (ELISA) (Elabscience, China, E-EL-R2466c).

### 2.6 Analysis of insulin secretory vesicles spatial distribution in INS-1 cells via transmission electron microscopy (TEM)

Insulin secretory vesicle density, number of plasma membrane-stopped vesicles, and other micro indexes were measured using TEM at 5, 10, 20, and 30 min after stimulation with KRBB buffer containing 50 mM glucose. The values of normal control, high glucose control, and high glucose +15% SQC groups were compared.

Briefly, after SQC treatments, INS-1 cells were fixed overnight using 2.5% glutaraldehyde in phosphate-buffered saline (PBS) at 4 °C, post-fixed with 1% osmium tetroxide for 2 h at room temperature. Then the fixed cells were dehydrated step by step in ethanol and acetone, embedded, and sectioned. The ultrastructure of the sections was then observed and imaged by TEM (Hitachi, Japan, HT7800). The images were analyzed using ImageJ processing.

### 2.7 Detection of NOD1 and RIP2 mRNA expression by real-time fluorescence quantitative reverse transcription polymerase chain reaction (RT-qPCR)

INS-1 cells (2 × 10^5^/well) were cultured in 6-well plates, pretreated with serum with or without ML130 (2μM, NOD1 inhibitor, MedChemExpress, USA, HY-18639) for 1 h, and then treated with 5.5 or 50 mM glucose for 24 h, followed by 15% SQC for another 24 h (25–27). Total cellular RNA was extracted using the Animal Total RNA Isolation Kit (Foregene, China, NO. RE-03014) and reverse transcribed into cDNA using 5 × All-In-One MasterMix (with AccuRT Genomic DNA Removal kit) (Abm, China, G492). Real-time qPCR was performed using BlasTaqTM 2 × qPCR MasterMix (Abm, China, G891) and a 20 μL reaction system (containing 2μL of cDNA). The reaction protocol was as follows: initial denaturation at 95 °C for 10 min, followed by 40 reaction cycles of 10 s denaturation at 95 °C and 30 s annealing at 60 °C. The primer sequences (Sangon Biotech, China) are detailed in Table 3. The relative expression of transcription was calculated using the 2^−Δ*ΔCT*^ method.

**Table 3:** Sequences of primers used in RT-qPCR.

**Gene**	**NCBI gene ID**	**Direction**	**Primer sequence (5^′^3^′^)**
NOD1	500,133	FORWARD	ATGCTTGTTCTGCCGACTGTAGTG
		REVERSE	GAGGTTGTTGTTGTCCAGGTCTAGG
RIP2	362,491	FORWARD	GCTCGTGCTCCTTGACTGTGATAAG
		REVERSE	GGATTGGTTCAGGCAGGCTTCAG
β-actin	81,822	FORWARD	TGTCACCAACTGGGACGATA
		REVERSE	GGGGTGTTGAAGGTCTCAAA

### 2.8 Detection of NOD1 and RIP2 protein expression by Western blot

The proteins were acquired by cell lysis using RIPA lysis buffer (Biosharp, China, BL504A) augmented with protease and phosphatase inhibitors (Servicebio, China, G2007-1ML). The acquired protein was then quantified using a BCA protein quantification kit (Biosharp, China, BL521A). Then, 20 μg of extracted protein was mixed with 5 × loading buffer, denatured at 95 °C for 5 min, separated by sodium dodecyl sulfate-polyacrylamide gel electrophoresis (SDS-PAGE), and transferred to the polyvinylidene fluoride (PVDF) membrane (MerckMillipore, USA, IPVH00010). The membranes were blocked with 5% skimmed milk for 1 h at room temperature, treated overnight with primary antibody [anti-NOD1 (1:1000, Cell Signaling Technology, USA, 3545S), anti-RIP2 (1:1000, Cell Signaling Technology, USA, 4142S), anti-β-actin (1:5000, Affinity, USA, AF7018)] at 4 °C, probed with secondary antibodies HRP-linked goat anti-rabbit IgG (1:10000, Multi Sciences, China, GAR0072) for 30 min at room temperature and visualized via the enhanced chemiluminescence (ECL) kit (Biosharp, China, BL523B). Protein blot images were captured using a chemiluminescence imaging system (CLINX, China, ChemiScope 6100), and analyzed via ChemiScope analysis software.

### 2.9 Statistical analysis

SPSS 26.0 and GraphPad Prism 8.0 software were employed for statistical analysis, and all the data were expressed as mean ± standard error of the mean (SEM) of at least 5 independent experiments. For multiple-group comparisons, one-way ANOVA or Welch's test with LSD or Tamhane's T2 *post hoc* tests were performed. *p* < 0.05 was considered statistically significant.

## 3 Results

### 3.1 SQC inhibits high glucose-induced INS-1 cell damage

**Figure 1:**
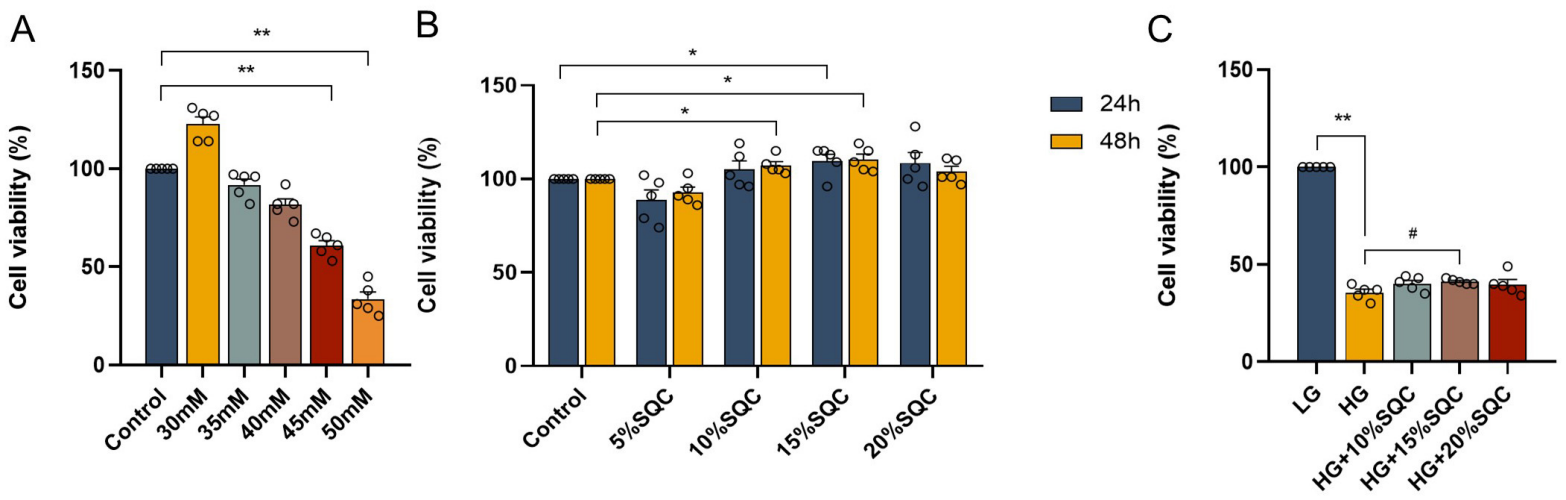
Effect of SQC on the viability of INS-1 cells induced by high glucose. **(A)** INS-1 cells were incubated with 30, 35, 40, 45, and 50 mM glucose for 24 h. Cell viability was assessed by CCK-8 assay. **(B)** INS-1 cells were treated with 5, 10, 15, and 20% SQC for 24 or 48 h, and their viability was assessed using a CCK-8 assay. **(C)** SQC had an inhibitory effect on 50 mM glucose-induced INS-1 cell damage. Untreated normal cells (control) were assigned 100% values. Data is expressed as mean ± SEM (*n* = 5). *p < 0.05 vs. control or LG group, **p < 0.01 vs. control or LG group, #*p* < 0.05 vs. HG group.

To determine the optimal conditions for high glucose induction, INS-1 cells were cultured in a medium containing high (30 to 50 mM concentrations) glucose for 24 h before their viability was measured (28). The results revealed that compared with the control group, INS-1 cells exposed to high glucose from 30 to 50 mM had decreased cell viability in a concentration-dependent manner (Figure 1A). Considering these findings together with prior experience in high-glucose modeling from previous cell-based experiments, a treatment regimen of 50 mM glucose for 24 h was selected as the optimal condition to induce hyperglycemic damage in subsequent experiments (29).

To select the appropriate concentration of SQC, cells were treated with different concentrations (5, 10, 15, and 20%) for 24 and 48 h before viability analysis. The results showed that 10–20% SQC did not affect cell viability (Figure 1B).

Furthermore, this study also investigated the protective effect of SQC against high glucose-induced INS-1 cells. Briefly, the cells were incubated with 5.5 or 50 mM glucose for 24 h and then treated with various SQC concentrations (10, 15, and 20%) for another 24 h. The results indicated that 50 mM glucose significantly reduced INS-1 cell viability; however, the viability of these cells was markedly improved after the treatment with 15% SQC (*p* = 0.032, Figure 1C). These data suggested that SQC has an inhibitory effect on high glucose-induced INS-1 cell damage.

### 3.2 SQC promotes insulin secretion in high glucose-induced INS-1 cells

**Figure 2:**
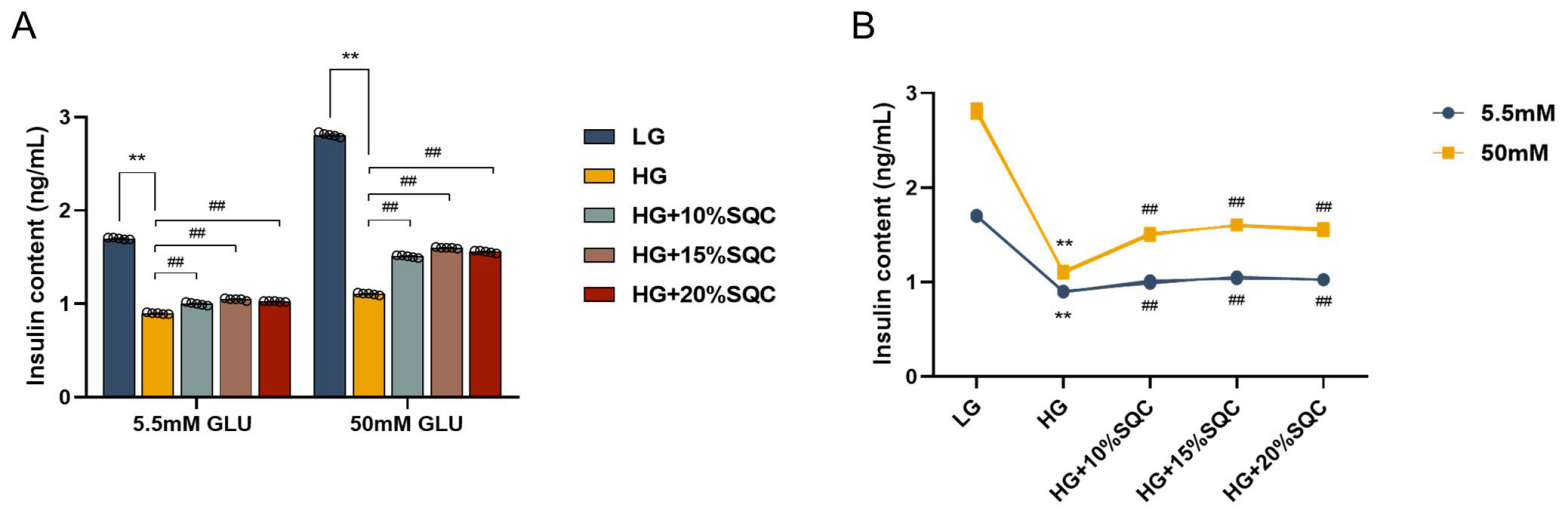
Effect of SQC on insulin secretion in high glucose-induced INS-1 cells. **(A, B)** INS-1 cells (5 × 10^3^/well) were cultured in 96-well plates containing 5.5 or 50 mM glucose for 24 h and then treated with SQC (10, 15, and 20%) for 24 h. The cells were incubated with Krebs-Ringer bicar-bonate HEPES buffer for 1 h, followed by stimulation with Krebs-Ringer buffer containing 5.5 or 50mM glucose for another 1 h. Insulin levels were assayed by ELISA assay. The normal and high glucose concentrations were set as 5.5 and 50 mM, respectively. Data is expressed as mean ± SEM (*n* = 5), **p < 0.01 vs. LG group, ##*p* < 0.01 vs. HG group.

To assess the impact of SQC on insulin secretion of pancreatic β-cells, the GSIS was performed. The INS-1 cells exposed to a 50 mM glucose condition exhibited a marked decrease in insulin secretion (all *p* < 0.0001, Figure 2A); however, SQC treatment improved this reduction. Furthermore, a concentration-dependent “inverted U-shaped” response was observed in 10, 15, and 20% SQC groups (all *p* < 0.0001, Figure 2B). Overall, these data indicate that SQC (at an optimal concentration of 15%) can improve insulin secretion in INS-1 cells under a high glucose condition.

### 3.3 SQC promotes the maturation and transport of insulin secretory vesicles in high glucose-cultured INS-1 cells

**Figure 3:**
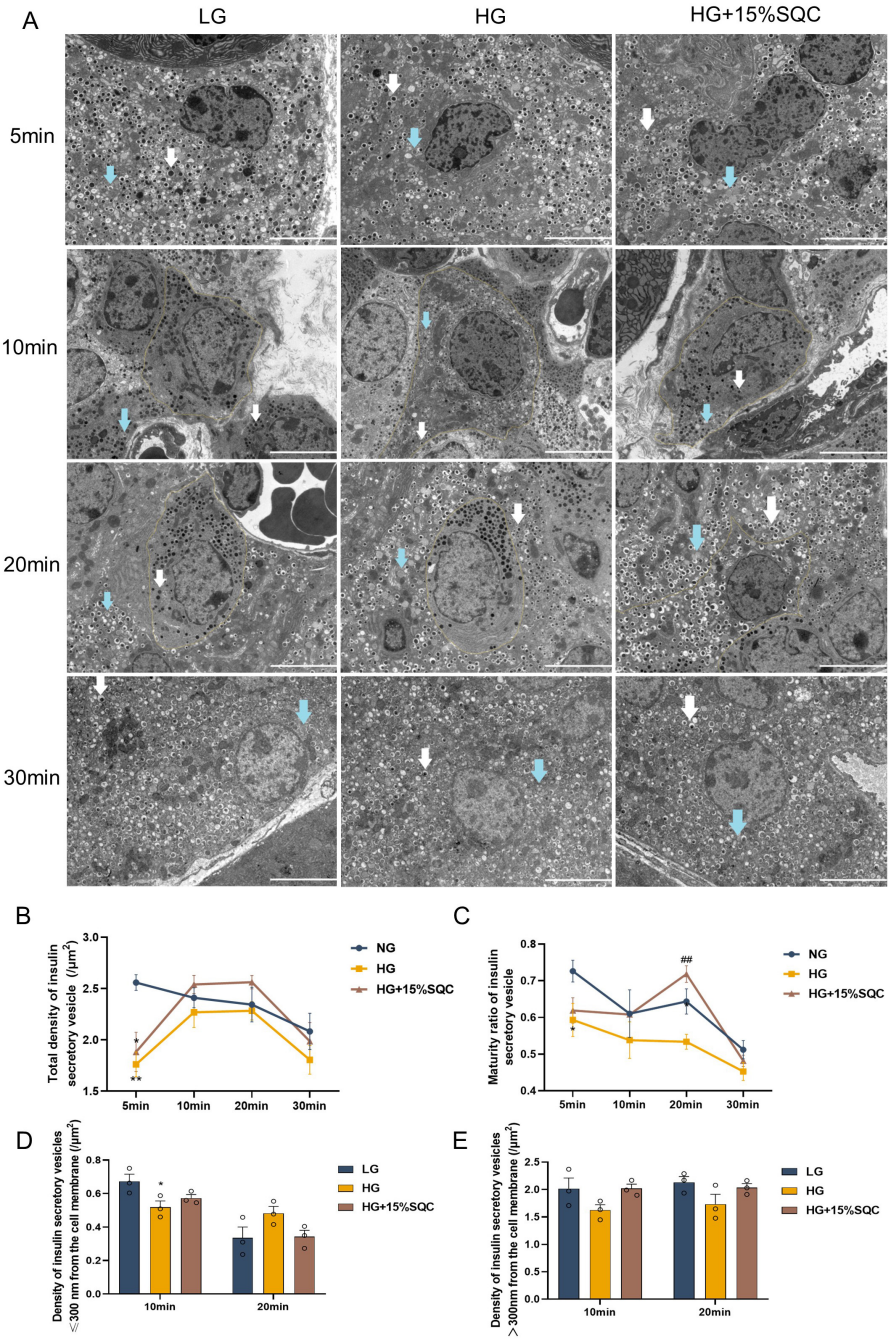
Effect of SQC on maturation and transport of insulin secretory vesicles. **(A)** TEM photomicrographs ( × 8.0 k) of the LG, HG, and HG + 15% SQC groups at 5, 10, 20, and 30 min after high glucose stimulation in GSIS. Scale, 5 μm. White arrow indicates mature insulin secretory vesicles, blue arrow denotes immature insulin secretory vesicles, and yellow line marks the cell membrane. **(B)** Total density of insulin secretory vesicles at each time point of the LG, HG, and HG + 15% SQC groups. **(C)** Maturity ratio of insulin secretory vesicles at each time point of the LG, HG, and HG + 15% SQC groups. **(D, E)** Vesicle density of the LG, HG, and HG + 15%SQC groups at different distances from the cell membrane after 10 and 20 min of high-glucose stimulation. Data is expressed as mean ± SEM (*n* = 3), *p < 0.05 vs. LG group, **p < 0.01 vs. LG group, ^##^*p* < 0.01 vs. HG group. Two researchers independently performed the analysis using ImageJ. Total density of insulin secretory vesicles was calculated as the total number of insulin secretory vesicles divided by the effective field area. Maturity ratio of insulin secretory vesicles was defined as the density of mature insulin secretory vesicles divided by the total density of insulin secretory vesicles. Density of insulin secretory vesicles within 300 nm of the cell membrane was calculated as the total number of insulin secretory vesicles within 300 nm of the cell membrane divided by the cell area. Density of insulin secretory vesicles located more than 300 nm from the cell membrane was calculated as the total number of such vesicles divided by the cell area. Inclusion criteria for distance measurement relative to the cell membrane: the center of the vesicle must be located within 300 nm of the boundary line.

To further validate the effect of SQC on the maturation and transport of insulin secretory vesicles in INS-1 cells, the insulin intracellular distribution and transport trend of insulin secretory vesicles at 5, 10, 20, and 30 min after high-glucose stimulation in GSIS were observed via TEM (Figure 3A). It was observed that initially, after the 15% SQC intervention, there was no increase in insulin secretory vesicles compared with the HG group, indicating that SQC potentially regulates the second phase of GSIS (10) (Figure 3B). Following a period of incubation, particularly after 20 min of stimulation with high glucose, 15% SQC increased both the total density (*p* = 0.143) and the maturity ratio (*p* = 0.003) of insulin secretory vesicles in INS-1 cells exposed to high-glucose-induced damage (Figures 3B, C). Moreover, by comparing the density of insulin secretory vesicles within a 300 nm range of the cell membrane across different groups at 10 and 20 min, we observed that in the 15% SQC group, vesicle density in this region increased at 10 min but decreased at 20 min, following a trend similar to that of the control group. This suggests that SQC facilitates the translocation of insulin secretory vesicles toward the plasma membrane (Figures 3D, E).

### 3.4 Effect of SQC on the NOD1/RIP2 signaling pathway in high glucose-induced INS-1 cells

**Figure 4:**
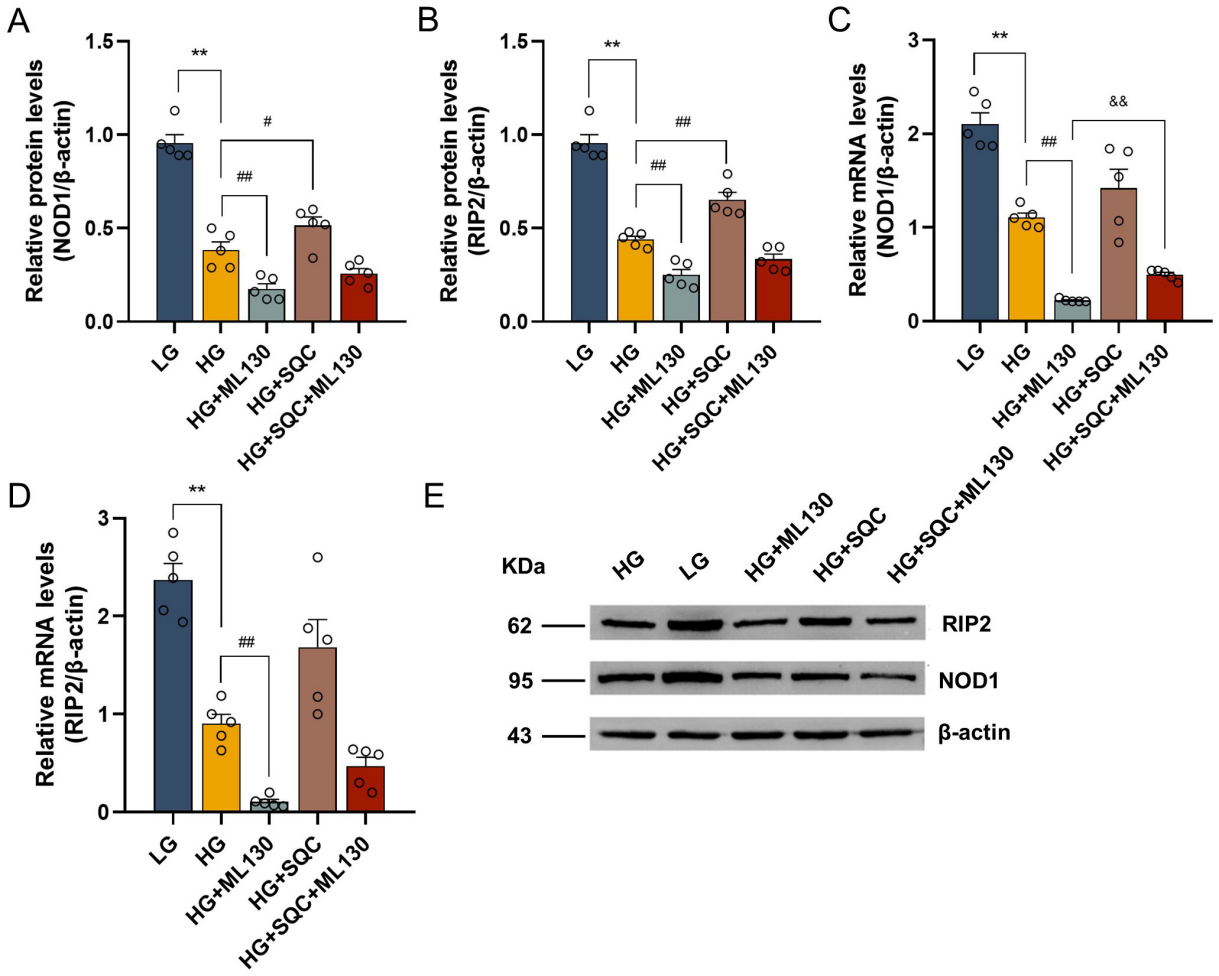
Effect of SQC on NOD1/RIP2 signaling pathway. INS-1 cells were pretreated with serum with or without ML130 for 1 h, followed by treatment with 5.5 or 50 mM glucose for 24 h, and then with 15% SQC for another 24 h. **(A, B, E)** The protein and **(C, D)** mRNA expression levels of NOD1 and RIP2 were assessed by Western blot and RT-qPCR, respectively. Data is expressed as mean ± SEM (*n* = 5), **p < 0.01 vs. LG group, ^#^*p* < 0.05 vs. HG group, ^##^*p* < 0.01 vs. HG group, ^&&^*p* < 0.01 vs. HG+ML130 group.

The effect of SQC on the NOD1/RIP2 signaling pathway was assessed by Western blot and RT-qPCR analyses. The results showed that high glucose decreased the expression of NOD1 and RIP2 protein (*p* < 0.0001, both; Figures 4A, B, E) and transcripts (*p* = 0.004 and *p* = 0.002, respectively; Figures 4C, D). However, 15% SQC intervention notably up-regulated the expressions of NOD1 and RIP2 protein (*p* = 0.029 and *p* < 0.0001, respectively; Figures 4A, B, E) and transcripts (*p* = 0.886 and *p* = 0.393, respectively; Figures 4C, D) after high-glucose induction. Moreover, ML130 was observed to reverse the activating effect of 15% SQC on the NOD1/RIP2 signaling pathway. Altogether, these data indicate that SQC can modulate the NOD1/RIP2 signaling pathway to mitigate cellular damage caused by high glucose levels.

## 4 Discussion

Inadequate insulin secretion is the predominant pathological cause of persistent hyperglycemia and glycaemic variability (30). Insulin secretion from the pancreatic β-cells is a complex and highly regulated process, which includes insulin biogenesis and stimulus-secretion coupling (31). Insulin is derived from preproinsulin, which is synthesized and converted into proinsulin in the rough endoplasmic reticulum. The proinsulin is then transported to the trans-Golgi, where it is sorted by clathrin and forms immature insulin secretory vesicles, which further mature by fusing with homotypic vesicles and acidification and are converted from proinsulin to insulin, which is then transported to the readily releasable pool or reserve pool for release (32). As the blood glucose concentration rises, glucose enters the cytoplasm of β-cells for metabolism, resulting in increased intracellular adenosine triphosphate (ATP) concentration, which can close ATP-sensitive potassium (KATP) channels, depolarize the β-cell membrane, and promote calcium influx. Elevation of cytoplasmic Ca^2+^ levels can trigger exocytosis to release insulin (33). The orderly maturation and transport of insulin secretory vesicles are the important basis for vesicle release and insulin secretion.

SQC has been used in China for over 20 years because of its beneficial effects against T2DM. Several studies have investigated its active ingredients and quality control (24, 34). SQC can preserve pancreatic islet function by modulating glucose homeostasis, restoring insulin sensitivity, reducing oxidative stress and inflammation in pancreatic tissues, modulating compensatory pancreatic β-cell proliferation, enhancing individual pancreatic β-cell secretion, and preventing pancreatic cell apoptosis (10). However, the stages and underlying mechanisms of SQC in stimulating insulin secretion remain unclear. Therefore, this study investigated the precise function of SQC in promoting insulin secretion at the functional, morphological, and molecular levels. At the functional level, cell viability and GSIS analyses revealed that under high glucose conditions, SQC could inhibit INS-1 cell injury and promote insulin secretion, which is consistent with previous findings (9, 10). Furthermore, TEM analysis assessed the total density, maturity ratio, intracellular distribution, and secretion trend of insulin secretory vesicles at different time points in GSIS. The results showed that, at the morphological level, SQC increased the total density of insulin secretory vesicles in hyperglycemic INS-1 cells. This suggests that more proinsulin is packed into the vesicles, providing a sufficient reserve for insulin secretion. In addition, SQC upregulated the maturity ratio of insulin secretory vesicles following high-glucose stimulation in GSIS, particularly at 20 min, indicating its potential to promote insulin conversion. Moreover, SQC also enhanced the density of insulin secretory vesicles within a 300 nm region adjacent to the cell membrane, supporting timely insulin exocytosis. Notably, SQC treatment shortened extracellular release time and improved the efficiency of vesicle transport to the cell membrane, enabling a faster response to elevated glucose levels and consequently enhancing glycemic regulation. Collectively, these findings indicated that SQC can enhance the storage and release efficiency of insulin, thereby ameliorating insulin secretion dysfunction.

At the molecular level, this beneficial phenomenon may be attributed to the regulation of the NOD1/RIP2 signaling pathway. Nucleotide-binding oligomerization domain-like receptors (NLRs) are a class of PRRs located in the cytoplasm, which play an essential role in innate immunity and inflammatory responses (35). Among these NLRs, NOD1 is the most prominent protein, which comprises three domains: a leucine-rich repeat (LRR) domain, a nucleotide-binding and oligomerization domain (NACHT), and a caspase recruitment domain (CARD) (36, 37). LRR domains recognize peptidoglycan fragment, iE-DAP, and can activate NOD1, which forms a CARD-CARD connection with downstream adaptor RIP2 (38, 39). The NOD1-RIP2 complex then recruits transforming growth factor-β-activated kinase-1 (TAK1) to activate NF-κB and MAPK signaling pathways, thereby promoting an inflammatory response that increases the expression of cytokines and chemokines (40, 41). In addition to its classical role as a bacterial sensor, NOD1 signaling can also exert a dual regulatory effect on modulating metabolic processes in the body (13). On the one hand, NOD1 recognizes a diverse array of pathogenic ligands, thereby mediating metabolic inflammation and inducing insulin resistance, lipolysis, adipose tissue, and liver inflammation, as well as participating in the pathogenesis of diabetes and its complications (16, 42–44). On the other hand, NOD1 not only promotes metabolic inflammation but also impacts normal endocrine function. It has been found that activating the NOD1/RIP2 pathway in pancreatic β-cells via the gut-pancreas axis can modulate insulin maturation and distribution, thereby promoting insulin secretion (19). Qin Zhang et al. confirmed that the intestinal-derived NOD1 ligand, peptidoglycan fragment, would enter the pancreas through systemic circulation, activating and binding to the NOD1 on pancreatic β-cells. Subsequently, NOD1 translocates to the surface of insulin secretory vesicles and recruits downstream adaptor RIP2. Both proteins colocalize at the vesicle to activate downstream signaling involving the small guanosine triphosphatase (GTPase) Rab1a, an essential component for insulin conversion and intracellular trafficking, thereby driving the maturation and intracellular transport of insulin and promoting insulin secretion (45–47). During normal conditions, this signal facilitates insulin secretory vesicles' processing and modification to form insulin, evenly distributes insulin in the cytoplasm, and promotes microtubule movement, which is necessary for appropriate insulin secretion. When the NOD1 ligand or NOD1/RIP2 is absent, the maturation and transport of insulin secretory vesicles are impaired, which causes abnormal cytoplasmic distribution of proinsulin and insulin, thereby affecting insulin secretion.

Based on this study and previous findings, it has been demonstrated that SQC can differentially regulate the NOD1/RIP2 signaling pathway in response to varying levels of NOD1 ligands in the body, thereby protecting the insulin-secreting function of pancreatic β-cells. In previous experiments, we observed that diabetic rats exhibited intestinal microbiota dysbiosis, intestinal mucosal injury, and an increase in Lysozyme, resulting in elevated levels of circulating iE-DAP. Excessive iE-DAP abnormally activated the NOD1/RIP2 signaling pathway in pancreatic β-cells, triggering immune-mediated inflammation and impairing pancreatic function. However, following SQC treatment, the composition of the gut microbiota was restored, the intestinal mucosa was repaired, circulating iE-DAP levels were reduced, and the activation of the NOD1/RIP2 and downstream MAPK pathways in pancreatic tissue was suppressed, thereby preserving the functional integrity of pancreatic β-cells (20). In this study, when the NOD1 ligand was absent, SQC was able to activate the NOD1/RIP2 signaling pathway in INS-1 cells under high glucose conditions and improve the maturation and transportation of insulin vesicles, thereby protecting their secretory function. For example, it was reported that *Astragalus*, the primary herb in SQC, and its active components can exert an activating effect comparable to that of the NOD1 agonist iE-DAP (48). This study verified the expression of NOD1 and RIP2 proteins and their transcripts in INS-1 cells by Western blot and RT-qPCR. The results demonstrated that SQC significantly up-regulated protein content of NOD1 and RIP2 under the high-glucose induction. Although the mRNA levels of NOD1 and RIP2 also exhibited an increasing trend following SQC intervention, the changes were not statistically significant. This observation may be attributed to temporal and spatial disparities between transcription and translation processes, or to the potential involvement of SQC in post-translational modification events. Overall, these data suggest that SQC modulates the expression of these two key molecules and activates downstream signaling events, thereby accelerating the maturation and transport of insulin secretory vesicles. This study contributes to a comprehensive understanding of the therapeutic potential of SQC in T2DM and may provide a theoretical basis for developing novel therapies capable of effectively regulating pancreatic β-cell function, thereby offering promise for improved treatment strategies and enhanced clinical outcomes in T2DM patients.

However, this study still has several limitations: (1) insulin secretion and intracellular transport of insulin vesicles were not examined in INS-1 cells incubated with NOD1 inhibitors, which prevented verification of the direct relationship between the “SQC–NOD1/RIP2–vesicle” pathway; (2) the effects of SQC on key downstream biomarkers of the NOD1/RIP2 signaling pathway, such as Rab1a, NF-κB, and MAPK, have not been comprehensively assessed; and (3) due to the significant biological differences between *in vitro* and *in vivo* conditions, it is necessary to apply diabetic germ-free animals to further verify the effect of SQC on the interaction between pancreatic NOD1/RIP2 and vesicle secretion in the absence of NOD1 ligands. These limitations will be addressed in future animal studies.

## 5 Conclusions

This study indicated that SQC exhibits protective effects on INS-1 cells against high glucose-induced injury. Furthermore, it enhances the maturation and transport of insulin secretory vesicles, thereby improving INS-1 cells' secretory function and benefiting pancreatic islet health. These benefits may be related to the activation of the NOD1/RIP2 signaling pathway. Overall, this study offers valuable insights into SQC's potential as a promising treatment for T2DM by targeting β-cell function.

## Data Availability

The original contributions presented in the study are included in the article/Supplementary material, further inquiries can be directed to the corresponding authors.

## References

[1] Zheng Y Ley SH Hu FB. Global aetiology and epidemiology of type 2 diabetes mellitus and its complications. Nat Rev Endocrinol. (2018) 14:88–98. doi: 10.1038/nrendo.2017.151 29219149

[2] Tomic D Shaw JE Magliano DJ. The burden and risks of emerging complications of diabetes mellitus. Nat Rev Endocrinol. (2022) 18:525–39. doi: 10.1038/s41574-022-00690-7 35668219 PMC9169030

[3] Hudish LI Reusch JE Sussel L. β Cell dysfunction during progression of metabolic syndrome to type 2 diabetes. J Clin Invest. (2019) 129:4001–8. doi: 10.1172/JCI129188 31424428 PMC6763241

[4] International Diabetes Federation. IDF Diabetes Atlas, 11th editon Brussels, Belgium: International Diabetes Federation (2025). Available online at: https://diabetesatlas.org/resources/idf-diabetes-atlas-2025/ (Accessed August 22, 2025).

[5] Weir GC Gaglia J Bonner-Weir S. Inadequate β-cell mass is essential for the pathogenesis of type 2 diabetes. Lancet Diabetes Endocrinol. (2020) 8:249–56. doi: 10.1016/S2213-8587(20)30022-X 32006519 PMC7098467

[6] Xie C. Construction of prevention and treatment system in TCM for diabetic macrovascular disease under guidance of incubative pathogen theory. J Bas Chin Med. (2022) 28:1205–9. doi: 10.19945/j.cnki.issn.1006-3250.2022.08.024

[7] Zhang X Liu Y Xiong D Xie C. Insulin combined with Chinese medicine improves glycemic outcome through multiple pathways in patients with type 2 diabetes mellitus. J Diabetes Investig. (2015) 6:708–15. doi: 10.1111/jdi.12352 26543546 PMC4627549

[8] Zhang X Wang H Fu X Xie C. Effect of Shenqi Compound recipe on damage of oxidative stress and nitration stress in rats of diabetic vascular disease. Chin Arch Tradit Chin Med. (2017) 35:2066–9. doi: 10.13193/j.issn.1673-7717.2017.08.038

[9] Liu Y Zhang X Chao J Wang H Wang J Xie C. Effect of Shenqi Compound on islet β-cell function in type 2 diabetic GK rats. Chin J Exp Tradit Med Formulae. (2020) 26:34–9. doi: 10.13422/j.cnki.syfjx.20202237

[10] Yang C Liu H Xie Z Yang Q Du L Xie C. The protective role of shenqi compound in type 2 diabetes: A comprehensive investigation of pancreatic β-cell function and mass. Biomed Pharmacother. (2023) 166:115287. doi: 10.1016/j.biopha.2023.115287 37572639

[11] Tang R Xie C Zhang X. NOD1: a metabolic modulator. Front Endocrinol. (2025) 15:1484829. doi: 10.3389/fendo.2024.1484829 39906040 PMC11790428

[12] Chen L Cao S-Q Lin Z-M He S-J and Zuo J-P. NOD-like receptors in autoimmune diseases. Acta Pharmacol Sin. (2021): 1742-56. doi: 10.1038/s41401-020-00603-2 PMC8564530 33589796

[13] Zangara MT Johnston I Johnson EE McDonald C. Mediators of metabolism: an unconventional role for NOD1 and NOD2. Int J Mol Sci. (2021) 22:1156. doi: 10.3390/ijms22031156 33503814 PMC7866072

[14] Schertzer JD Tamrakar AK Magalhaes JG Pereira S Bilan PJ Fullerton MD . NOD1 activators link innate immunity to insulin resistance. Diabetes. (2011) 60:2206–15. doi: 10.2337/db11-0004 21715553 PMC3161332

[15] Shiny A Regin B Balachandar V Gokulakrishnan K Mohan V Babu S . Convergence of innate immunity and insulin resistance as evidenced by increased nucleotide oligomerization domain (NOD) expression and signaling in monocytes from patients with type 2 diabetes. Cytokine. (2013) 64:564–70. doi: 10.1016/j.cyto.2013.08.003 24018334 PMC4158007

[16] Chan KL Tam TH Boroumand P Prescott D Costford SR Escalante NK . Circulating NOD1 activators and hematopoietic NOD1 contribute to metabolic inflammation and insulin resistance. Cell Rep. (2017) 18:2415–26. doi: 10.1016/j.celrep.2017.02.027 28273456

[17] Huang W Gou F Long Y Li Y Feng H Zhang Q . High glucose and lipopolysaccharide activate NOD1- RICK-NF-κB inflammatory signaling in mesangial cells. Exp Clin Endocrinol Diabetes. (2016) 124:512–7. doi: 10.1055/s-0042-105641 27169686

[18] Val-Blasco A Prieto P Gonzalez-Ramos S Benito G Vallejo-Cremades MT Pacheco I . NOD1 activation in cardiac fibroblasts induces myocardial fibrosis in a murine model of type 2 diabetes. Biochem J. (2017) 474:399–410. doi: 10.1042/BCJ20160556 27803247

[19] Zhang Q Pan Y Zeng BH Zheng XJ Wang HF Shen XY . Intestinal lysozyme liberates Nod1 ligands from microbes to direct insulin trafficking in pancreatic beta cells. Cell Res. (2019) 29:516–32. doi: 10.1038/s41422-019-0190-3 31201384 PMC6796897

[20] Wang X. Mechanism study on the improvement of blood glucose fluctuation in T2DM mice through regulation of intestinalflora and NOD1 protein by Shenqi compound prescription. Chengdu: Chengdu university of traditional Chinese medicine. (2024).

[21] Maggi CA Meli A. Suitability of urethane anesthesia for physiopharmacological investigations in various systems. Part 1: General considerations Experientia. (1986) 42:109–14. doi: 10.1007/BF01952426 2868911

[22] Qin M-Y Huang S-Q Zou X-Q Zhong X-B Yang Y-F Zhang Y-T . Drug-containing serum of rhubarb-astragalus capsule inhibits the epithelial-mesenchymal transformation of HK-2 by downregulating TGF-β1/p38MAPK/Smad2/3 pathway. J Ethnopharmacol. (2021) 280:114414. doi: 10.1016/j.jep.2021.114414 34314804

[23] Lin T Zhang W Zhang Y Sun X Huo C He T . Screening methods for optimal serum concentration of Chinese medicine:a review. Chin J Exp Tradit Med Form. (2023) 29:195–202.

[24] Zhang C Bi L Gao H Xie C Fu X Liu S . Fingerprint study of the Shenqi compound extract by UHPLC. Chin Meas Test. (2021) 47:54–61.

[25] Jiang HY Najmeh S Martel G MacFadden-Murphy E Farias R Savage P . Activation of the pattern recognition receptor NOD1 augments colon cancer metastasis. Protein Cell. (2020) 11:187–201. doi: 10.1007/s13238-019-00687-5 31956962 PMC7026222

[26] Pena-Ponce MGd Jimenez MT Hansen LM Solnick JV Miller LA. The Helicobacter pylori type IV secretion system promotes IL-8 synthesis in a model of pediatric airway epithelium via p38 MAP kinase. PLoS One. (2017) 12: e0183324. doi: 10.1371/journal.pone.0183324 28813514 PMC5557493

[27] Wang M Ye X Hu J Zhao Q Lv B Ma W . NOD1/RIP2 signalling enhances the microglia-driven inflammatory response and undergoes crosstalk with inflammatory cytokines to exacerbate brain damage following intracerebral haemorrhage in mice. J Neuroinflammation. (2020) 17:364. doi: 10.1186/s12974-020-02015-9 33261639 PMC7708246

[28] Ganesan K Ramkumar KM Xu B. Vitexin restores pancreatic β-cell function and insulin signaling through Nrf2 and NF-κB signaling pathways. Eur J Pharmacol. (2020) 888:173606. doi: 10.1016/j.ejphar.2020.173606 32980348

[29] Xing Y He Y Zhang Y Wang H Peng S Xie C . Emodin Alleviates High-Glucose-Induced Pancreatic β-Cell Pyroptosis by Inhibiting NLRP3/GSDMD Signaling. Evid Based Complement Alternat Med. (2022) 2022:5276832. doi: 10.1155/2022/5276832 35265148 PMC8898799

[30] Rorsman P Renström E. Insulin granule dynamics in pancreatic beta cells. Diabetologia. (2003) 46:1029–45. doi: 10.1007/s00125-003-1153-1 12879249

[31] Hou JC Min L Pessin JE. Insulin granule biogenesis, trafficking and exocytosis. Vitam Horm. (2009) 80:473–506. doi: 10.1016/S0083-6729(08)00616-X 19251047 PMC4324607

[32] Omar-Hmeadi M Idevall-Hagren O. Insulin granule biogenesis and exocytosis. Cell Mol Life Sci. (2021) 78:1957–70. doi: 10.1007/s00018-020-03688-4 33146746 PMC7966131

[33] Vakilian M Tahamtani Y Ghaedi K A. review on insulin trafficking and exocytosis. Gene. (2019) 706:52–61. doi: 10.1016/j.gene.2019.04.063 31039435

[34] Shan L Yuan C Yang H Cao L Tan J Gao T . Simultaneous determination of saponins in Shenqi compound granules by HPLC-ELSD. Chin J Exp Tradit Med Formulae. (2016) 22:102–5. doi: 10.13422/j.cnki.syfjx.2016140102

[35] Carneiro LA Magalhaes JG Tattoli I Philpott DJ Travassos LH. Nod-like proteins in inflammation and disease. J Pathol. (2008) 214:136–48. doi: 10.1002/path.2271 18161746

[36] Fernández-García V González-Ramos S Martín-Sanz P. García-del Portillo F, Laparra JM, and Boscá L. NOD1 in the interplay between microbiota and gastrointestinal immune adaptations. Pharmacol Res. (2021) 171:105775. doi: 10.1016/j.phrs.2021.105775 34273489

[37] Ohto U. Activation and regulation mechanisms of NOD-like receptors based on structural biology. Front Immunol. (2022) 13:953530. doi: 10.3389/fimmu.2022.953530 36189327 PMC9520476

[38] Moreno L Gatheral T. Therapeutic targeting of NOD1 receptors. Br J Pharmacol. (2013) 170:475–85. doi: 10.1111/bph.12300 23848281 PMC3791987

[39] Bauer S Hezinger L Rexhepi F Ramanathan S Kufer TA. NOD-like receptors-emerging links to obesity and associated morbidities. Int J Mol Sci. (2023) 24:8595. doi: 10.3390/ijms24108595 37239938 PMC10218625

[40] Kobayashi K. Naohiro Inohara, Hernandez LD, Galán JE, Núñez G, Janeway CA, et al. RICK/Rip2/CARDIAK mediates signalling for receptors of the innate and adaptive immune systems. Nature. (2002) 416:194–9. doi: 10.1038/416194a 11894098

[41] Nembrini C Kisielow J Shamshiev AT Tortola L Coyle AJ Kopf M . The kinase activity of Rip2 determines its stability and consequently Nod1- and Nod2-mediated immune responses. J Biol Chem. (2009) 284:19183–8. doi: 10.1074/jbc.M109.006353 19473975 PMC2740541

[42] Rivers SL Klip A Giacca A. NOD1: an interface between innate immunity and insulin resistance. Endocrinology. (2019) 160:1021–30. doi: 10.1210/en.2018-01061 30807635 PMC6477778

[43] Rodrigues E-Lacerda R Fang H Robin N Bhatwa A Marko DM Schertzer JD. Microbiota and Nod-like receptors balance inflammation and metabolism during obesity and diabetes. Biomed J. (2023) 46: 100610. doi: 10.1016/j.bj.2023.100610 37263539 PMC10505681

[44] Apaza CJ Días M Tejedor AG Boscá L Llopis JM. Contribution of nucleotide-binding oligomerization domain-like (NOD) receptors to the immune and metabolic health. Biomedicines. (2024) 12:341. doi: 10.3390/biomedicines12020341 38397943 PMC10886542

[45] Hickey AJ Bradley JW Skea GL Middleditch MJ Buchanan CM Phillips ARJ . Proteins associated with immunopurified granules from a model pancreatic islet beta-cell system: proteomic snapshot of an endocrine secretory granule. J Proteome Res. (2009) 8:178–86. doi: 10.1021/pr800675k 19055480

[46] Hutagalung AH Novick PJ. Role of Rab GTPases in membrane traffic and cell physiology. Physiol Rev. (2011) 91:119–49. doi: 10.1152/physrev.00059.2009 21248164 PMC3710122

[47] Mukhopadhyay A Nieves E Che FY Wang J Jin LJ Murray JW . Proteomic analysis of endocytic vesicles: Rab1a regulates motility of early endocytic vesicles. J Cell Sci. (2011) 124:765–75. doi: 10.1242/jcs.079020 21303926 PMC3039020

[48] Koehler H Puchalski K Ruiz G Jacobs B Langland J. The role of endophytic/epiphytic bacterial constituents in the immunostimulatory activity of the botanical, Astragalus membranaceus. Yale J Biol Med. (2020) 93:239–50. Available online at: https://pmc.ncbi.nlm.nih.gov/articles/PMC7309664/ 32607085 PMC7309664

