# Supplementary material for: Shenqi compound enhances pancreatic β-cell secretion by promoting the maturation and transport of insulin secretory vesicles through the NOD1/RIP2 signaling pathway
Source: Front Nutr. 2026 Jan 26;12:1690849. doi: 10.3389/fnut.2025.1690849 (PMC12884057; doi:10.3389/fnut.2025.1690849)
Supplement: Supplementary file 2 [file Image_1.pdf]

Supplementary Material

1 Supplementary Figures

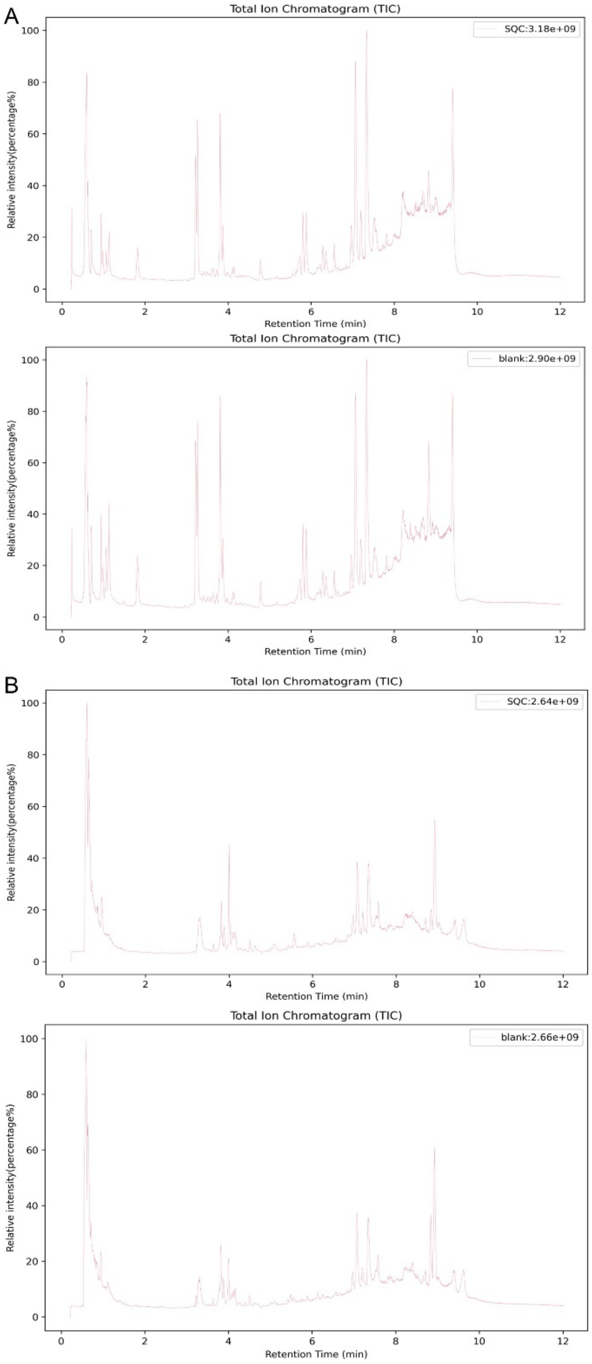

Supplementary Figure S1. The total ion current chromatogram of SQC-serum and blank-serum by UPLC-MS. (A) The total ion current of SQC-serum and blank-serum analyzed in positive-ion mode. (B) The total ion current of SQC-serum and blank-serum analyzed in negative-ion mode

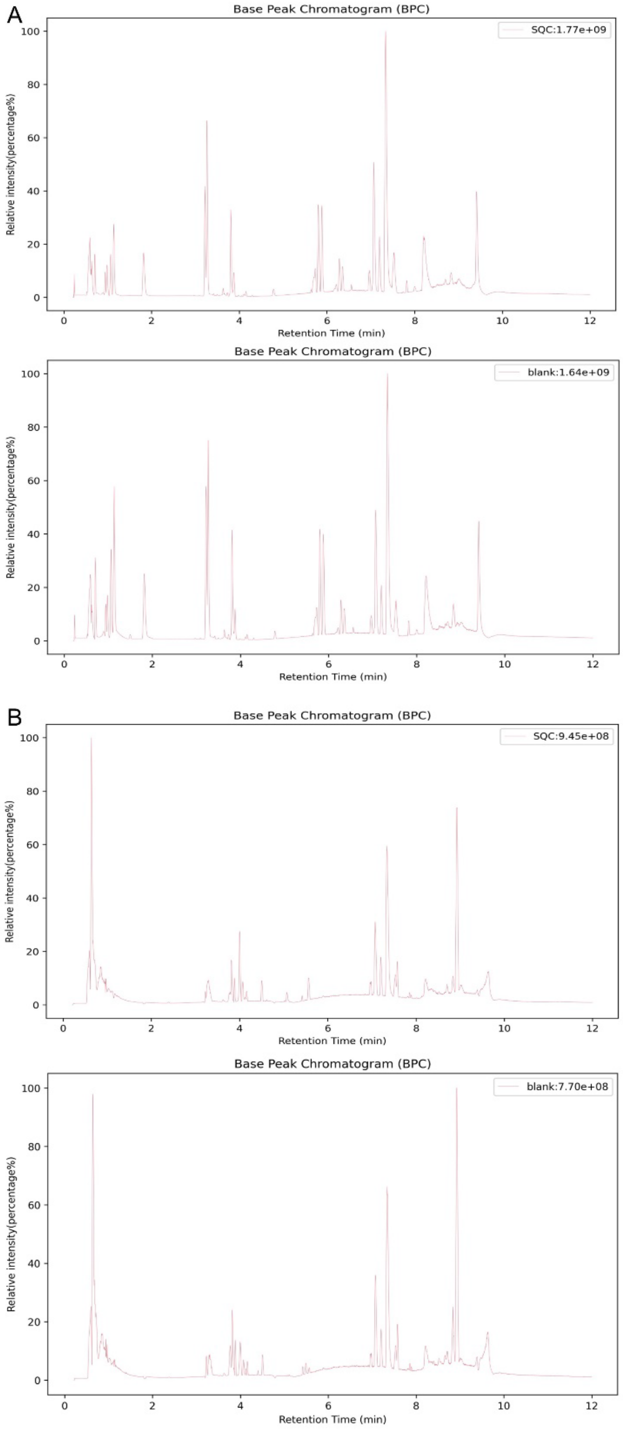

Supplementary Figure S2. The base peak chromatogram of SQC-serum and blank-serum by UPLC-MS. (A) The base peak intensity of SQC-serum and blank-serum analyzed in positive-ion mode. (B) The base peak intensity of SQC-serum and blank-serum analyzed in negative-ion mode.
